# PAX-MAC: A Low Latency Anycast Protocol with Advanced Preamble †

**DOI:** 10.3390/s20010250

**Published:** 2020-01-01

**Authors:** Tales Heimfarth, João Carlos Giacomin, Edison Pignaton de Freitas, Gustavo Figueiredo Araujo, João Paulo de Araujo

**Affiliations:** 1 Department of Computer Science, Universidade Federal de Lavras, 37200-900 Lavras, Brazil; giacomin@ufla.br (J.C.G.); kustavo@gmail.com (G.F.A.); 2 Institute of Informatics, Universidade Federal do Rio Grande do Sul, 90040-060 Porto Alegre, Brazil; edison.pignaton@inf.ufrgs.br; 3 Centre National de la Recherche Scientifique, Sorbonne University, LIP6/DELYS, France; joao.araujo@lip6.fr

**Keywords:** anycast, MAC protocol, wireless sensor networks

## Abstract

Wireless sensor networks employ duty-cycles to save energy, with the cost of enlargement of end-to-end latency. Cross-layer protocols that use anycast medium access control achieve latency reduction in asynchronous duty-cycled wireless sensor networks (WSNs). A series of strobed preambles is sent in order to achieve rendezvous with the next relay, selected from a forwarding candidate set (FCS). This paper proposes PAX-MAC: Pramble Ahead Cross-layer Medium Access Control. It is a novel anycast protocol for low latency packet propagation in duty-cycled WSNs. In PAX-MAC, preambles propagate ahead of data packet, prospecting the route towards sink node, while the message is sent some hops later. Simultaneous propagation of preambles and data packets provides latency reduction. The cardinality of FCS determines the average preamble propagation speed, which is lower bounded by data packet propagation speed. Differently from other approaches, our protocol takes the data packet size into account in order to maintain an optimal distance between preamble and data to minimize latency. For determining this distance, a detailed mathematical model is introduced. The performance of several state-of-the-art asynchronous protocols was appraised and compared with PAX-MAC. Our protocol outperforms in latency all other protocols for the simulated scenarios. Its energy expenditure was compatible with the best result among the other protocols. In the worst case, PAX-MAC spent 6% more energy than the best one for a gain of 20% in latency.

## 1. Introduction

Advances in wireless sensor networks (WSNs) technology enable a number of emerging applications that were not even thought years ago. WSNs are composed of small components, denominated “sensor nodes”, which work cooperatively to achieve the desired functionalities of environmental/event surveillance or monitoring. They are in general dense networks and the sensor nodes are randomly deployed in the environment. This characteristic gives them high robustness, since alternative redundant communication paths are provided by the number of sensor nodes that cover a common area [1]. Sensor nodes are expected to operate for a long time without human intervention, hence all the actions must be taken in the most energy-efficient fashion. This is the first concern while developing software for WSN, including networking protocols.

The main strategy to save energy and extend network lifetime is to reduce radio usage, since it is the main energy consumer of a sensor node. The amount of data transferred between nodes is very small in WSNs, then the radios are off almost all the time and are turned on for small intervals to receive and transmit data only. Some position estimation algorithm is employed in order to the nodes estimate their relative position, so that a geographic routing protocol can be used. This approach reduces the coordination effort to maintain updated knowledge of the network topology [2]. Medium access control (MAC) layer protocols manage the duty-cycling [3] of nodes, switching its state from sleep to awake and back to sleep. Duty-cycle is small (<10%) since the sleep state is very longer than awake state. All actions, measurements, processing, and communication, are made during the small awake state. Normally all the nodes in the network adopt the same duty-cycle, but synchronization is not always required. The use of duty-cycles increases the latency per hop due to the sleep-delay problem. A node that is going to send a message towards a destination must wait until the relay node (i.e., the next-hop node) is awake before sending the message [4]. Some authors propose to stagger the wake up time of nodes in the path towards destination as strategy to overcome the sleep-delay problem [5,6,7,8,9,10,11,12]. Even though this is the most effective method to reduce the end-to-end latency in duty-cycled WSN, all the proposals face the difficulty to keep synchronism among the sensor nodes.

Another approach to mitigate the sleep-delay problem is the use of anycast protocols. Anycast is a communication pattern which exploits path redundancy available in dense wireless networks [4,13,14,15]. Normally, anycast based MACs interact with routing protocols, in a cross-layer approach. Decisions about the next relay in the route towards the destination node are taken considering information from both layers. The cross-layer design explores the possibility to retrieve and/or change parameters of two or more layers in order to achieve an optimization objective [1,16,17]. Anycast protocols normally unify functionalities of asynchronous MAC, as X-MAC [3], with a geographic routing protocol, as presented in [4,18]. The final decision about the next node to relay a data packet is taken in MAC layer. Among the nodes targeted in anycast communication, the first one to wake up and respond to the preamble is elected the next relay.

This work proposes PAX-MAC, Preamble ahead cross-layer MAC protocol. This asynchronous protocol employs some strategies to reduce end-to-end latency in duty-cycled WSNs. Anycast communication pattern reduces the time needed to achieve rendezvous with a next relay node. Preambles propagate ahead separated from data, in such a way that both packets advance in distinct points of the path towards destination, simultaneously. Preambles establish communication between relay nodes and stagger their wake up times in order to speed up data propagation. Differently from state-of-the-art approaches, PAX-MAC does not require energy to keep synchronism as [6,19,20], and, at the same time, it is not restricted to one direction traffic as [8] nor to burst traffic as [7]. PAX-MAC achieves similar latency of a network without duty-cycling, maintaining the energy profile of protocols that use sleep/awake cycles. This is accomplished without any costly inter-node synchronization.

The seminal ideas of this protocol were first introduced in [21]. The published article was an incomplete initial proposal since parameters of the protocol were selected empirically, collisions were not handled properly and the evaluation performed was not extensive. In the current article, a novel mathematical model to support optimal protocol parameters decision was introduced. In addition, a mechanism to handle possible collisions between packets of different transmission flows is also included. A much more extensive assessment of our protocol was accomplished, demonstrating its superior performance when compared to state-of-the-art. The results were obtained in scenarios with single transmission and environments with multiple concurrent data flows.

Besides this introduction, the rest of this paper is organized: Section 2 discusses related works, presenting an extended literature review that provides the reader with a consistent base for comparisons. Section 3 exposes the principles of Anycast Asynchronous MAC. The protocol is presented in Section 4. Section 5 provides details of the mathematical model describing the rationale behind PAX-MAC proposal. Section 6 presents the experiments performed by means of simulations and the acquired results. Conclusions and discussions of future works are presented in Section 7.

## 2. Related Work

In WSNs, communication between a source (S) and a destination (D) node is normally realized by multi-hop due to the limited transmission range, requiring a routing mechanism. In this process, several relay nodes are responsible to receive and send data packets further. This task must be executed with a reduced energy cost.

Medium access and routing protocols are at the core of multi-hop communication. Enhancements in energy efficiency of these two protocols may enlarge the life-time of a sensor network considerably. MAC protocols have to coordinate access to the medium maintaining very low energy consumption. On its turn, network protocols aim at reducing the communication latency in low power WSNs.

According to [22,23] there are two main approaches to develop MAC protocols for WSNs: contention-based and reservation-based approaches. Reservation-based approaches (like time division multiple access - TDMA - protocols) is less attractive due to its strict synchronization requirements, demanding large overhead and restricting its scalability. Contention-based MAC protocols can be divided in asynchronous, represented by the canonical Berkely-MAC (B-MAC) [24], and those with common active period, as the canonical sensor-MAC (S-MAC) [25].

In order to save energy, sensor nodes remain in low energy consumption state, called sleep mode, most of the time (>90%). The sensor nodes are periodically reactivated (awake) to perform data acquisition, computations and data communication during a short slot of time and then return to the low power (sleep) mode. MAC protocols are responsible to control this duty-cycled operation. In common active period approaches, a loose synchronization is required, so that all the nodes in the network wake up almost at the same time to execute those tasks before going back to sleep. With asynchronous protocols, all the nodes use the same duty-cycle, with the same sleep and awake intervals, but the wake-up times of the nodes are independent from each other. The awake interval is very shorter compared to the previous approach, which results in lower energy consumption in asynchronous networks. On the other hand, the MAC layer must have a mechanism to establish communication between nodes.

The usage of duty-cycles is the best way to save energy and enlarge lifetime [5,26] of whole WSN. On the other hand, it increases end-to-end latency due to sleep-delay [11]. When a sensor node has a message to transmit, it must wait until the receiver stays awake and ready to receive. Several duty-cycled MAC protocols were proposed aiming to achieve minimal delays and high energy efficiency. Staggering the awake times of all nodes along the path towards destination is the best choice to reduce sleep-delay [5,6,7,8,9,10,11,12,19,20]. In this way, data can be forwarded in a pipelined fashion, coordinating the waiting time of all nodes in the path towards destination. In DMAC [8] nodes do not keep a common sleep/awake scale as in S-MAC, but use a staggered wake-up schedule in the sink’s direction. The nodes closer to the sink (downstream) wake up a bit later than the nodes more distant (upstream). ADC-MAC [12] and Joint Routing and MAC Protocol (JRAM) [11] were inspired in DMAC. ADC-MAC is adaptable to traffic and distance to sink node by configuring the duty-cycle. JRAM is a protocol designed to support multiple sink nodes and keeps its synchronism using a special packet SYNC as the S-MAC. Express-MAC (EX-MAC) [7] is an asynchronous duty-cycled protocol designed for event detection applications. Differently from DMAC, it does not maintain synchronization between nodes. Transmitter nodes with EX-MAC send a preamble signal in order to achieve rendezvous with a targeted receiver, in a similar manner of B-MAC. EX-MAC sends a sequence of strobed short preambles as those encountered in X-MAC [3] protocol.

EX-MAC was designed for WSNs dedicated to detecting events. Such networks remain inactive most of the time and have high activity periods when events of interest occur and messages are sent towards the sink. The first event message propagates towards the sink node in the same way of the asynchronous X-MAC, with a small difference, EX-MAC staggers the awake times of the nodes involved in the event report. When subsequent events are detected, communication latency is drastically reduced due to the alignment of the sensor nodes that are at the transmission path, as well as DMAC. The latency reduction occurs only in the transmission of burst events.

Some novel MAC protocols were inspired in EX-MAC. DuoMAC [5] is a protocol adaptable to the type of traffic (real-time and non real-time). When the traffic is non real-time, its behavior mimics X-MAC protocol with a low duty-cycle. For real-time traffic, high duty-cycle with pipelined fashion transmissions is employed. RM-MAC [10] is an asynchronous received initiated approach adaptable to the traffic pattern. When transmissions are frequent, the nodes are staggered and transmissions are made in pipeline as EX-MAC.

Another strategy to reduce sleep-delay in duty-cycled WSNs is to separate data from control messages. Control messages, as preambles and RTS (Request To Send), are transmitted ahead, in order to establish communication and reserve the channel for data transmission. RMAC - Routing-Enhanced MAC [6] keeps a common scale of sleep/awake, in the same way of S-MAC [25]. Differently from S-MAC, RMAC sends data messages separately from control messages. During the awake period, PION (PIONeer control frame) messages are sent several hops ahead. The communication channel is reserved for data transmission during the common sleep period. In the sleep time, only those nodes that will participate in the communication wake up in the scheduled time to receive and send data. Experimental results from performed simulations presented a 6-times reduction in the latency of RMAC compared to S-MAC. RMAC inspired other proposals, as those presented in [19,20].

Increased energy savings have been obtained with cross-layered WSNs. In the cross-layer approach, decisions about the way each layer will behave are taken jointly, though considering information coming from other layers [1]. A great number of cross-layer proposals have been dedicated to the interaction of network (NET), medium access (MAC) and physical (PHY) layers [5,9,10,11,12,16,17]. Interactions between a geographical routing (NET) with a preamble-based MAC protocols result in a special cross-layer group denominated anycast [4,13,14,27,28,29].

**Figure 1:**
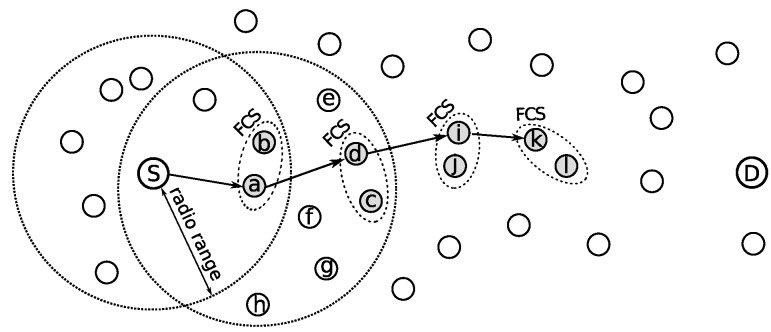
Principle of the anycast MAC protocols.

Anycast protocols take advantage of the path redundancy, i.e., multiple next-hop candidate nodes available in the network. Differently from the traditional unicast protocols, there is no unique choice of a relay node, but a subset of neighbors, denominated forwarding candidate set (FCS), with appropriate conditions to forward a message, as depicted in Figure 1. In this example, FCS has two members, represented by gray circles. Sender node starts to transmit a series of short preambles, in the same way of X-MAC [3]. The first FCS member that wakes up replies to the preamble packet and assumes the relay function. Therefore, communication is established more quickly, reducing the latency caused by sleep-delay.

GeRaF (Geographic Random Forwarding) [29] was a pioneer anycast protocol aiming at reducing the latency in asynchronous protocols using probing. Sender node considers as FCS members all neighbors that give to the message advancement towards destination node. If node *a* is the sender in Figure 1, the neighbors *c*, *d*, *e*, *f* and *g* will compose its FCS. Members closer to destination (*D*) are given higher priority to forward data message.

Convergent MAC (CMAC) [28] is an improvement of GeRaF. CMAC defines a minimum advancement required ($r_{0}$) as a criteria to select the members of FCS. The neighbors of the sender that do not provide an advancement towards the destination greater than $r_{0}$, are not allowed to reply to the preamble, even though one of them wakes up before a FCS member. Figure 1 can represent a network using CMAC with $r_{0}$ defined as 60% of radio range. If node *a* is the sender, then only neighbors *c* ad *d* will take part in FCS. The authors consider negligible the length of data packets.

Restricting the number of members in FCS, with CMAC, the sender would take a longer time to receive an answer to the preambles than it would take with GeRaF, but the advancement of the data packet would be larger, since only neighbors closer to destination (*D*) are allowed to forward messages. The selection of $r_{0}$, in CMAC, depends on traffic load and the network density. Data length is not taken into account. If the traffic is heavy, the $r_{0}$ is reduced in order to include more nodes in the FCS and reduce the advance time.

Another cross-layer protocol inspired in GeRaF is AGA-MAC (Adaptive Geographic Anycast Medium Access Control) [18]. Following the same principle of CMAC, a threshold in the advancement towards the destination is imposed. In AGA-MAC, this threshold depends on data length. If data payload is short, threshold is reduced in order to reduce advance time. If data payload is long, threshold is enlarged in order to reduce the hops necessary to reach destination. This was the first paper addressing the influence of data packet length in the latency of WSNs that use anycast protocols. The authors demonstrated that there is an optimum threshold value for each data packet length that provides the smallest latency in the network.

A cross-layer proposal called Any-MAC (Anycast MAC) [4] considers existing NET and MAC protocols with small modifications in both of them. Only asynchronous MAC protocols are considered. Any-MACs of the probe-based type use MAC protocols that perform periodic channel sampling to identify transmissions in the neighborhood as the X-MAC [3]. Hybrid asynchronous protocols use both wake-up schedule exchange and probing. The latency reduction is obtained following the same opportunistic routing principle used by GeRaF [29]. The FCS is determined in the sender’s network layer according to the calculated routing cost for each neighbor. The network layer informs the FCS to the MAC layer. The first FCS member that replies to the sender signal is elected the next-hop node. A greater number of FCS members results in a lower average time to data packets to proceed forwarding. However, as negative effect, there is an increment in the chance of collisions in sending answer signals when two or more FCS members wake up in time to listen and reply to the preamble. The authors present simulation results for the probe-based Any-MAC using an extension of the X-MAC protocol with two and three members in FCS. An average latency reduction of 50% was obtained by Any-MAC in comparison to the X-MAC protocol.

The proposed PAX-MAC belongs to the group of anycast protocols following the principle of opportunistic routing as GeRaF. Besides that, PAX-MAC incorporates the staggering method to perform fast data packet transmission and the advanced sending of preambles used by RMAC. The result is a cross-layer protocol involving routing and MAC for duty-cycled asynchronous WSNs with enhanced efficiency compared to the previous approaches. Our protocol presents low latency results, close to networks without duty-cycles for a diverse data packet lengths.

## 3. Anycast Asynchronous MAC

The anycast strategy is employed in asynchronous MAC protocols in order to reduce the sleep-delay caused by the independent sleep/awake cycles of each node. This class of protocols combines MAC layer and Network layer functions, exploring concepts of geographical routing combined with a preamble sampling strategy to ensure that a receiver will be awake in order to receive data. The concept of anycast is to explore the inherent redundancy in the network, i.e., multiple next-hop candidate nodes available in the network, reducing sleep-latency. Differently from unicast, this is accomplished by opportunistically forwarding data packets to a next hop selected from a group of possible relays (forwarding candidate set (FCS)), as depicted in Figure 1. Nodes have no information about wake-up schedules of the neighbors.

In an anycast asynchronous MAC, a sensor node with data to transmit first sends a series of short preambles in order to rendezvous with an awakening relay. After each preamble the sender settles the radio in the reception mode and waits during an interval for an answer. Neighboring nodes are performing the sleep/awake cycles, i.e., they only wake up and check whether the channel is busy. Occasionally, a member of the FCS will wake up and notice the presence of a preamble in the channel. This node answers to the preamble with an early-acknowledgment (eACK) signal in order to interrupt the preambles sequence and establish communication.

Design decisions of an anycast protocol are the FCS cardinality, its selection criteria and the definition of which node is responsible for the choice. The FCS cardinality has consequences in the end-to-end latency of a packet. A higher number of nodes in the FCS leads to, on average, smaller response times, reducing the one-hop latency. Nevertheless, the average size of the hop is reduced, increasing the number of hops spent to route the message to destination, which may increase the total latency. For different packet sizes and network densities, an optimal FCS cardinality can be employed to accomplish the minimal total latency. This phenomenon was studied in our previous work [18].

The criteria to select the FCS members is related to, for example, distance to final destination, battery residual energy and node connectivity. Most anycast asynchronous MAC employ as selection metric the advancement towards destination, i.e., how much closer to destination the next relay is.

The decision whether a given node is included in the FCS may be realized in the sender or receiver side. When the sender is responsible to select this group, it must know the position and other relevant state information from the neighboring nodes (as in [4,14,15,30]). Based on that, the FCS members are selected and their identifiers are informed in the preamble messages. When the decision is taken in the receiver side, the sender transmits in the preamble the necessary parameters to support the decision process. Each potential next-hop decides whether it is candidate to relay the packet or not based on the received information (as in [1,27,28,29]).

## 4. The PAX-MAC Protocol

In this section, our novel protocol is presented in details. The cross-layer nature of PAX-MAC comes from the interaction of network and MAC layers. Final routing decisions are taken in MAC layer, according to parameters provided by NET layer and current local conditions of WSN.

### 4.1. Overview

PAX-MAC combines a geographical routing with an anycast based protocol. Geographical routing means that a packet is relayed based on the position of the nodes, being a stateless protocol. An example of geographical routing is the GPSR (Greedy Perimeter Stateless Routing) [31], where, in greedy mode, the node nearest to the destination is chosen as next hop. Since our protocol is anycast, the next relay is selected from a set of candidates (FCS). This set is comprised by nodes which gave the greatest advancement towards destination. For that, the relay node is aware of neighboring positions. In the case of PAX-MAC, the destination is not a specific node ID but the node that is nearest to a given destination position. For the sake of simplicity, in this paper, we will call this node as destination *D*.

An example of application suitable to our protocol is the control of a oil refinement station. Lets suppose that wireless sensors are installed on the pipes, in order to detect liquid leaks. Instead of each sensor sending the flow information to a central control, in order to calculate whether the inflow from various pipes sum up and check with the outflow, nodes can exchange information themselves in order to detect pipe leaks and raise an alarm when necessary. In this manner, a node positioned in a outflow pipe may send messages to input positions, to verify updated flows. The nodes nearest to these positions can answer the request, and for that they can use the position provided by the initiator of the procedure. Fast responses are important in such an environment.

Since time is a concern to many applications, the main goal of PAX-MAC is to reduce the high latency present in duty-cycled WSNs caused by sleep-delay. In order to save energy, all the nodes in the network adopt the same cycle interval, but their awake times are not synchronized. When a sender node has a message to transmit, it starts sending a series of short preambles to rendezvous with a relay node. Sender node interleaves the preambles with short hearing intervals to wait for an eACK packet. Besides the use of anycast communication pattern, PAX-MAC manages to further reduce the latency transmitting data packet separately from the preambles.

Differently from other anycast protocols, PAX-MAC does not send data just after receiving the eACK signal. Preamble packet is propagated ahead by relay nodes and is used as a way to perform channel reservation for data transmission. Data transmission is started only a predefined interval after the preamble. The idea of PAX-MAC is to send the preambles ahead in order to wake up the relay nodes and to reserve the communication channel for the time when data packets will be sent. Preambles carry the information about the time in which the data will be transmitted from one relay to the next. This time is incremented by a data transmission time each hop. This way, the preambles stagger the time the relays will wake up in order to transmit data. At the same time of data transmission, preambles are scheduling the next relays some hops ahead. This process is denominated split phase transmission.

In the first phase, preambles propagate asynchronously hop-by-hop, while in second phase, data travels through the established route in a synchronous form. Preamble propagation speed is not constant, even though its average speed is made equal to data propagation speed. Therefore, it is possible that data transmissions take place close to preambles, occurring a packet collision. To avoid collisions between preamble and data packets, source node must separate the transmissions of these packets by a suitable amount of time. This is accomplished by an initial delay before sending data. This time should be long enough in order to avoid collisions between data and preamble in a given node in the path, but it should not be too long, otherwise it would increase the overall latency.

**Figure 2:**
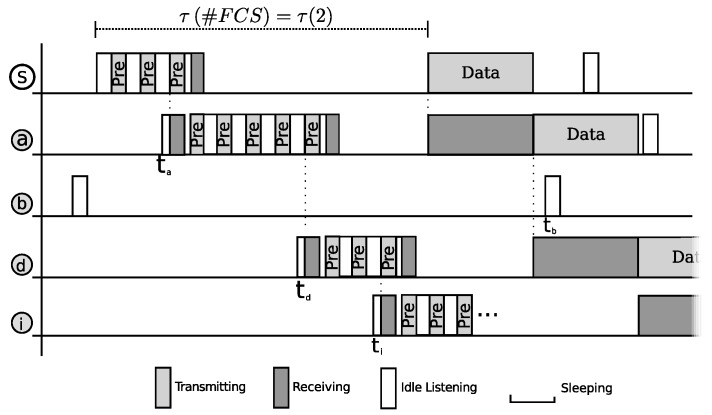
Overview of the PAX-MAC protocol.

As already presented, Figure 1 outlines a sample transmission of an anycast protocol. In PAX-MAC context, the split phase transmission process can be exemplified by this Figure combined with Figure 2. In our example, the source node *S* sends a data packet to a destination *D*. The transmission is depicted partially by both Figures (four hops). In this example, FCS cardinality employed was two. The packet received from upper layer engenders the transmission of a series of preambles in the source node, interleaved with listening periods for eACK reception. PAX-MAC does not define a special eACK packet as CMAC [28] and X-MAC [3]. When a FCS member wakes up and receives a preamble, it starts to send its own series in order to probe for the next hop. The first of these preambles is understood by the sender as an eACK signal, making it stop transmissions.

The FCS of the source node *S* comprises nodes *a* and *b* (Figure 1). The time line presented in Figure 2 shows that, after *S* started to send the series of preambles, node *a* woke up in $t_{a}$. This is before node *b*, which would wake up at $t_{b}$. In an anycast protocol such as PAX-MAC, the first node from the FCS which receives the preamble assumes the role of next relay towards destination *D*. Immediately, it begins to send a new series of preambles in order to signal its intention to transmit to a relay node of its own FCS (nodes *c* and *d*). The first preamble also acts as eACK for node *S*. At the same time, node *a* is scheduled to wake up immediately before the source node *S* starts to send data, in the future.

This process continues as presented in Figure 1. Node *d* becomes the next relay, after node *a*, as soon as it wakes up earlier than node *c*. Due to the asynchronous nature of our MAC protocol, the number of preambles spent in each hop to achieve rendezvous with the next relay may vary, as presented in Figure 2. In the sequence, node *d* starts to send preambles and programs its time to receive data packet. As explained, the first preamble is used as eACK in node *a*. After three preambles, node *i* wakes up and assumes the role of the next relay, starting its own preamble sequence. While node *i* is transmitting its preambles, node *S* undertakes the transmission of data packet to node *a*, which has just woken up to receive data. From here on, a simultaneous transmission of packet and preambles takes place. It is important to remark that these communications do not interfere with each other, in view of the distance held between the two transmitting nodes. Care is taken by the source node to delay data packet delivery by a time long enough to ensure its transmission takes place in a region far from preambles, with a distance larger than two times the radio range. The process of sending preambles ahead and data packet some hops behind continues until reaching destination node *D*.

It is important to note that we are not considering collisions of other data transmissions, we analyze the behavior of just one data sending at a time. We consider that all sender nodes encounter at least one neighbor to forward the packet in destination direction. If this condition fails, another algorithms must be used, as the right-hand rule [31].

In order to avoid collisions between both the packets, the propagation speed of preambles must be, on average, at least the same of data. The average time necessary to find an awake node among the selected set is adjusted accordingly with FCS cardinality. Thus, the number of FCS members depends on data packet size, such that, the smaller is data size, the larger is FCS cardinality. The average distance between the node sending data packet and the one transmitting the series of preambles is constant. Our protocol identifies imminent collisions due to deviations in preamble speed and manages to avoid them.

### 4.2. FCS Selection

PAX-MAC uses anycast communication pattern to speed up preamble advancement. If a small data packet has to be transmitted, more nodes must be included in FCS to reduce the mean time spent to establish preamble communication between the sender node and the next relay node. In other words, FCS cardinality has to be adjusted so that the average speed of preamble propagation will be at least the same as the data packet. Among the nodes that promote any advancement towards the destination, the best choices to compose FCS are those that can provide the greatest advancement. This guidance will reduce the total number of hops to reach the destination. As a consequence, less energy will be spent in this data transmission. The average preamble propagation speed is closely related to the number *r* of preambles sent until the sender node receives an eACK, which depends on *v*, the number of FCS members, as demonstrated by Equation (1):

(1) $$r\left(v\right)=\sum_{i=1}^{N_{p}}{\left(\frac{i}{N_{p}}\right)}^{v}.$$

$N_{p}$ is the maximum number of preambles to be sent in a cycle time. In our protocol, the time to transmit these preambles must be less or equal than the data packet transmission time. Then, Equation (1) can be used to obtain FCS cardinality in order to fit the average number of preambles (r(v)).

In our protocol, the decision to include nodes in the FCS is taken in the sender side. So, during each hop, the sender is aware of its own position, its neighbors’ positions and the position of the destination. Then, the sender will determine which neighbors will take part of the FCS. This information will be included in the preambles that will be sent. Only members of the FCS are allowed to answer with an eACK upon preamble reception.

### 4.3. Imminent Collision

A collision occurs when two or more radio messages sent in the same communication channel reach a receiver node, resulting in signal jamming. As a consequence, the receiving node can not decode any of the messages. For example, in Figure 2, if node *i* had waken up later than $t_{i}$ and node *d* would have to send 3 more preambles, a collision would occur in node *a*, since it would hear the preamble from node *d* while it was receiving data packet from source node *S*. PAX-MAC employs two mechanisms to avoid collision. The first one is the delayed delivery of data packet, in order to maintain a secure distance between the regions where both messages, data and preambles, take place. The second one is “imminent collision” detection. The node transmitting preambles can identify when a collision is about to happen.

PAX-MAC proposes the identification of this imminent collision and a mechanism to handle it so that it can be avoided. The current relay node, when sending preamble in order to contact the next relay, is aware of the data packet schedule of the previous relay nodes. When an imminent collision is identified, i.e., the current relay perceives that the next preamble to be sent is about to collide with the data coming from the previous relay, it suspends the preamble transmission. The current relay will wait for the data reception and only then it restarts preamble transmissions, acting as the source node (the first sender).

Following the previous example, consider node *d* is sending preambles when source node *S* is about to start sending data to node *a*. In this case, node *d* ceases the transmissions and waits until it receives the data packet from node *a*. So, node *d* must wait for a time corresponding to two data transmission intervals. After receiving data packet, node *d* starts to send preambles, and schedules the data release time based on the waiting time (τ), in the same way of the source node *S*.

The smaller the waiting time τ (in the source node), the shorter is the latency. However, smaller waiting times increase the chance of an imminent collision event. As a consequence, the overhead of restarting the transmission process increases the latency. The challenge then is to find the optimum waiting time in the source node.

The optimum waiting time is calculated in Section 6.2.1. This is the average number of hops separating preambles and data, which is expected to be less than ten. The node sending preambles must consider a guard interval to detect imminent collisions, in order to compensate the clock drift. low-power clocks employ 32 kHz tuning fork crystals, which are susceptible to significant drift over the industrial temperature range $-40^{{}^{\circ}}$ to $+85^{{}^{\circ}}$. Its drift can reach values as low as −200 ppm (parts per million) [32]. It means that in the worst case, the guard interval can reach $0.002t_{data}$, or 0.2% of the interval needed to transmit a data packet. If a temperature compensated resonator is used, a clock drift of ±50 ppm is verified, resulting in a lower guard interval.

### 4.4. Protocol State Machine

**Figure 3:**
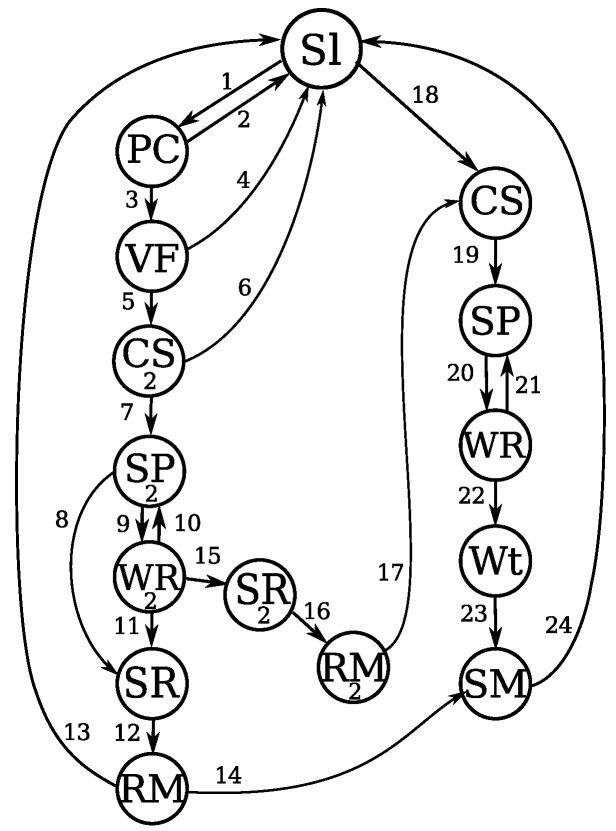
PAX-MAC state machine.

This section describes the state machine of PAX-MAC protocol as illustrated in Figure 3. Table 1 and Table 2 respectively present the meanings of the states and of the transitions.

Most of the time, when there is not any message being transmitted in the network, nodes repeat sleep/awake cycle. Each node toggles between states Sl (sleeping) and PC (probing channel) through transitions 1 and 2. The duty-cycle determines the probing time in relation to the total cycle.

In the case of a message coming from upper layers, a new transmission is started, initiating with a CS (carrier sensing) state (transition 18). If the channel is clear, there is not any transmission taking place in the communication channel near the sender node, transition 19 changes to the SP (sending preamble) state. In this state, a preamble packet is transmitted in broadcast. By transition 20, the state is changed to WR (waiting for a reply). In this state, the MAC protocol waits for an answer from a neighbor node belonging to the FCS. When an FCS member wakes up and receives the preamble, it starts to send its own preambles ahead. The first preamble functions as an eACK packet to previous node. If there is no FCS member awake to reply with a preamble packet, the transition 21 returns the protocol to the SP state. If a reply message is received, the transition 22 leads the protocol to the state Wt (waiting τ). In this state, the source node waits a computed time to send a data packet, according to the FCS cardinality. The optimal value for τ is determined employing the mathematical model presented in Section 5. This time is necessary to avoid collisions between the control packets (preambles) and the data packet. After this, transition 23 leads source node to the SM (sending message) state. By the end of the transmission, the protocol returns to the Sl state by transition 24.

When there is no message to be sent, the protocol alternates between states Sl and PC, as already explained. When a preamble is detected in the PC state, the MAC enters the state VF by the transition 3. In this state, the node that has received the preamble verifies whether it belongs to the FCS of the sender node. If the receiver is not a FCS member, it returns to Sl (by transition 4). All the nodes that receive a preamble and are not members of FCS, are not allowed to answer to other preambles, in order to avoid collisions with concurrent traffic. These nodes will remain in this state until data packet crosses the region. In this way, a possible concurrent traffic is prevented from interfering with this transmission. If the receiver node is an FCS member, the state switches to CS2 by the transition 5. In this state, the sender node puts the radio in listen mode to detect any transmitting signal in the communication channel. If a signal is detected, the node goes to Sleep state Sl through transition 6 to try a new transmission later. If the channel is empty, transition 7 takes the node to SP2. If this node is the final destination *D* of the message, it sends back a preamble packet with an empty FCS, just to signal the reception. After this, it goes to SR state by transition 8 and waits for data message. If this node is not the final destination *D*, it assumes the function of the current relay and starts to send its own preambles in order to find the next relay in the route. The first preamble sent by current relay acts at the same time as an eACK for the previous relay. After sending a preamble, the protocol takes the transition 9 and changes to WR2 state to wait for a reply from one of its FCS members. It is important to highlight that states SP and SP2 are similar in what they perform, but in the first one, the protocol is sending a starting message while the second one is reached during the relaying between intermediary nodes. When a node is waiting for a reply, in WR2 state, if no signal is detected in the channel after a specific amount of time, the protocol returns to SP2, by transition 10. If a reply is received, transition 11 drives the protocol to the state SR. In this state, the radio is deactivated until the instant of receiving data packet. When the waiting time is elapsed, the protocol reaches RM state by transition 12. In this state, data packet is received by the sensor node. After this reception, the final destination address is verified. If the message is addressed to the receiving node, the packet is sent to the upper layer and the protocol returns to the sleep state by transition 13. If the receiving node is not the destination *D*, the message has to be transmitted to the next relay node. Thus, the protocol reaches the state SM by transition 14. After data transmission, the sending node enters the Sl state through transition 24 and goes back to the sleep/awake cycle.

When an imminent collision is identified in WR2 state, the transition 15 drives the state machine to SR2 state in order to avoid collision between data and preamble packets in the upstream relay. In this state, the node interrupts the transmission of preambles and awaits the arrival of the data packet. Transition 16 leads the protocol to the state RM2 in which the data message is received. After this, the relay assumes the role of initiator *S*, and goes to the state CS by transition 17 and the communication process is restarted. Since preambles were interrupted, the transition 17 is responsible for restart their transmissions in the same way it was done in the source node.

## 5. Mathematical Model

In this section, we present an analytical model that enables the determination of optimal period τ. This is an interval between the time when sender node starts to send preambles and the time it releases data packet. This interval is necessary to avoid imminent collision. In order to calculate τ we have to know the probability of using *k* preambles to achieve an awake node in the hop *n*. This is the probability of avoiding an imminent collision. *k* must comprise a total time smaller than the one employed by the data message to achieve hop (n−3). Our mathematical model uses a large number of variables. In order to facilitate the reading task, Table 3 describe them.

### 5.1. Probability of Using k Preambles

The number *k* of preambles spent until an awake node is encountered in hop *n* is the sum of preambles used in each hop. So the probability of using *k* preambles depends on the probability of using a certain number *i* of preambles per hop. The probability function of using *i* preambles to encounter an awake FCS member in the next hop is given by:

(2) $$q\left(v,i\right)=\frac{{\left(N_{p}-i+1\right)}^{v}-{\left(N_{p}-i\right)}^{v}}{{N_{p}}^{v}},$$

where *v* is the cardinality of the FCS and $N_{p}$ is the maximum amount of preambles in a cycle time.

This formulation comes from the question: what is the probability of the sender to obtain an answer after preamble *i* when the number of FCS members is *v*? It is the sum of all combinations of answers of all the *v* FCS members only after the preamble *i*. In other words, none of the FCS members answered the sender before the preamble i+1. All of them would wake up and hear the preamble numbered from i+1 to $N_{p}$. This probability is calculated as:

(3) $$q^{\prime }\left(v,i\right)=\frac{{\left(N_{p}-i\right)}^{v}}{{N_{p}}^{v}}.$$

Using this result, we can calculate the probability of obtaining an answer exactly on preamble *i*, given by the difference:

(4) $$q\left(v,i\right)=q^{\prime }\left(v,i-1\right)-q^{\prime }\left(v,i\right)$$

which gives Equation (2).

The probability of spending *k* preambles until a sensor node wakes up in hop *n* is the combination of the probabilities q(v,i) of all hops, from 1 to *n*. For example, suppose a sensor network that uses a cycle time (the sleep/awake cycle) with the same duration of 3 cycles of sending preamble and waiting eACK ($N_{p}=3$). If we want to know the probability of achieving an awake FCS member in the fourth hop, using 6 preambles (k=6) since the first one in the sender node, we have to consider all combinations of sent preambles (*i*) in all four hops that totalize 6: (1,1,1,3); (1,1,2,2); (1,1,3,1); (1,2,1,2); (1,2,2,1); (1,3,1,1); (2,1,1,2); (2,1,2,1); (2,2,1,1) or (3,1,1,1). Then we have to compute the sum of probabilities of all these combinations. This calculation can be made recursively as explained bellow.

Equation (5) extends q(v,i) to embrace *n* hops. The probability of advancing *n* hops with *k* preambles (FCS with cardinality *v*) is given by the multi-part recursive equation:

(5) $$p\left(v,k,n\right)=\{\begin{array}{ll} 1 & \mathrm{if}\;n=0\;\mathrm{and}\;k=0\\ 0 & \mathrm{if}\;n=0\;\mathrm{and}\;k\neq 0\\ 0 & \mathrm{if}\;n\neq 0\;\mathrm{and}\;k\leq 0\\ \sum_{i=1}^{N_{p}}q\left(v,i\right)\cdot p\left(v,k-i,n-1\right) & \mathrm{otherwise} \end{array}$$

Equation (5) is derived from (2). The probability of advancing 0 hops with 0 preambles is 1. The probability of using *k* preambles to advance 0 hops is 0, whereas the probability of advancing n≠0 hops using 0 or less preambles is also 0.

In the other cases, Equation (5) returns the sum of all probabilities of using different combinations of preambles in the different hops to achieve hop *n*. A restriction is that the sum of preambles used in the hops $h_{0}$, $h_{1}$, …, $h_{n}$ must be *k*.

The first part of the sum (q(v,i)) returns the probability of using *i* preambles in the last hop, whereas the recursive part ($p\left(v,k-i,n-1\right)$) calculates the probability of using k−i preambles in all previous hops in combination. This is done for all possible amounts of preambles in the last hop, i.e., from i=1 to $i=N_{p}$.

### 5.2. Average Number of Preambles

As stated in Section 4.2, the average number of preambles necessary to encounter an awake node in the FCS, in each hop is given by Equation (1):

$$r\left(v\right)=\sum_{i=1}^{N_{p}}{\left(\frac{i}{N_{p}}\right)}^{v}.$$

This equation can be explained as following: in order to calculate the average number of preambles, a sum of all preamble numbers *i* multiplied by the probability of obtaining an answer in that preamble q(v,i) is performed, as detailed in Equation (6).

(6) $$\begin{array}{cc} r(v) & =\sum_{i=1}^{N_{p}}i\cdot q\left(v,i\right)\\ \; & =\frac{1}{{N_{p}}^{v}}\cdot \{\left[{\left(N_{p}\right)}^{v}-{\left(N_{p}-1\right)}^{v}\right]+\\ \; & +2\cdot \left[{\left(N_{p}-1\right)}^{v}-{\left(N_{p}-2\right)}^{v}\right]+\\ \; & +3\cdot \left[{\left(N_{p}-2\right)}^{v}-{\left(N_{p}-3\right)}^{v}\right]+\\ \; & \;\vdots \\ \; & +\left(N_{p}-1\right)\cdot \left[2^{v}-1^{v}\right]+\left(N_{p}\right)\cdot \left[1^{v}\right]\}\\ \; & =\frac{1}{{N_{p}}^{v}}\cdot \left\{{N_{p}}^{v}+{\left(N_{p}-1\right)}^{v}+{\left(N_{p}-2\right)}^{v}...+2^{v}+1^{v}\right\}=\sum_{i=1}^{N_{p}}{\left(\frac{i}{N_{p}}\right)}^{v}. \end{array}$$

### 5.3. Probability of Successful Transmission of n Hops

In order to calculate the delay time to release the data packet, it is important to estimate the probability of transmitting preambles successfully through *n* hops. This probability is evaluated in Equation (7). This means that, with probability of $ps_{n}\left(\delta ,v\right)$, no imminent collision event occurs during the transmission. δ is the initial delay to release data packet. This delay is calculated as a number of preamble propagation time (average value). Since τ is given in seconds, the relation between τ and δ is given by $\tau =\delta \cdot r\left(v\right)\cdot \left(t_{eACK}+t_{pre}\right)$. The preamble length is given by $t_{pre}$ and $t_{eACK}$ is the remission interval to wait for an eACK message. Intervals are given in seconds. *n* is the target number of hops.

(7) $$ps_{n}\left(\delta ,v\right)=\sum_{k_{3}=1}^{\delta \cdot r(v)}\sum_{\begin{array}{c} k_{4}=1\\ R_{4} \end{array}}^{N_{p}}...\sum_{\begin{array}{c} k_{n}=1\\ R_{n} \end{array}}^{N_{p}}p\left(v,k_{3},3\right)\prod_{b=4}^{n}p\left(v,k_{b},1\right).$$

$$R_{j}:\left(\delta +j-3\right)\cdot r\left(v\right)\geq \sum_{i=3}^{j}k_{i}.$$

The probability of transmitting the packet *n* hops without collision is calculated based on p(v,k,n), which is the probability of using *k* preambles to encounter an awake FCS member in all *n* hops, i.e., to deliver the preamble *n*-hops successfully. The main idea of Equation (7) is to sum the probability of successful transmission for all combinations of the number of preambles used in each hop. A successful transmission is characterized by a fast enough preamble advancement. This avoids collision with the data packet.

The transposition of the first three hops must be realized in at most δ·r(v) preambles to avoid collision since the data packet is released after this period. This is modeled by the first summation ($k_{3}$). Here $p(v,k_{3},3)$ is used, which is the probability of using $k_{3}$ preambles to overcome three hops. When considering the fourth hop, a restriction $R_{4}$ in the number of preambles ($k_{4}$) must be satisfied. The total number of preambles that may be employed to wake up the four first hops can not exceed (δ+1)·r(v). This is the sum of the data release time with the time necessary for transmitting the packet one hop further. The probability of transposing the three first hops is multiplied by the probability of transposing the fourth at the right number of preambles ($p(v,k_{4},1)$). This pattern is followed by the next hops (δ+2)·r(v), (δ+3)·r(v), etc. The restrictions are generalized as $R_{j}$. Summarizing, Equation (7) takes into account that no collision can happen in any intermediary hop, i.e., all steps must have successful transmission to result in a *n* hop collision-free transmission.

As already stated, with this equation, it is possible to calculate the probability of transmitting a packet *n* hops without restarting due to imminent collision. If the recovery mechanism is not implemented, it is possible to select an initial delay to release the data packet (τ) that avoids collisions altogether with a desired probability.

### 5.4. Optimal Initial Delay to Release Data Packet for n Hops

In this section, the formulation of an optimization problem to find the optimal initial delay to release the data packet is presented. Equation (8) presents the optimization problem for *n* hops. tl(n) is the average total latency, measured in data packet length, in the optimal case, to relay a packet *n* hops using our MAC scheme. It is the sum of the time spent by the packet to be transmitted *n* hops plus additional latency brought by the reservation of the channel and possible collisions (e(n)). $\delta_{n}$ is the initial delay to release the packet (this time interval is measured in data packet length), which should be optimized. ph(i,δ) is the probability of colliding (imminent collision) on hop *i* when the initial delay δ is employed.

(8) $$\begin{array}{cc} \; & tl(n)=e(n)+n\\ \; & e\left(n\right)=\min_{\delta_{n}\in R+}\;\left(\delta_{n}+\sum_{i=1}^{n-1}ph\left(i,\delta_{n}\right)\cdot e\left(n-i\right)\right)\\ \; & ph\left(i,\delta \right)=ps_{i}\left(\delta ,v\right)-ps_{i+1}\left(\delta ,v\right). \end{array}$$

The idea of the equation is to minimize the latency by means of selecting the appropriate initial delay for the current situation, i.e., the expected number of hops remaining to the packet destination. The expected latency is given by the transmission of the packet through *n* hops added to the protocol overhead e(n). This overhead comprises of the initial delay ($\delta_{n}$) to release data packet for *n* hops, in addition to the probability of imminent collision in each hop multiplied by the overhead caused by the event. The probability of imminent collision in the hop *i* is given by ph(i,δ). It is important to remark that this is a recursive optimization function, since for each imminent collision the whole process starts over. In this case, an optimal overhead is calculated again for the remaining path using the same method (recursive call to the minimization function e(n)).

For example, with n=10, we are calculating the protocol overhead in terms of latency for ten hops. This is the result of the initial delay $\delta_{n}$ for releasing the data packet added to the probability of imminent collision after the first transmission multiplied by the overhead caused by this event. In addition, all other possible imminent collisions (in the second relay, third, etc) should be also considered.

### 5.5. Average Advancement

In the last section, the optimal initial delay to release data packet for a given number of hops was determined. Since the positions of source (*S*) and destination (*D*) of the packet are known, the source can estimate the number of hops necessary to reach the final destination, employing the formulation presented in this section. For that, a mathematical model for the average advancement accomplished per hop is presented. When a relay node achieve rendezvous with one of its FCS members, the FCS member starts to send its own preambles. We say preambles make a “progress”. Since the FCS member is closer to destination *D* than the current relay, we say the preamble takes an advancement. The advancement is the difference between the distance measured from the relay nodes to destination (*D*).

Equation (9) presents the average advancement in each hop for a FCS cardinality of *v* nodes.

(9) $${adv}_{v}=\frac{1}{v}\sum_{m=1}^{v}a_{m},$$

where $a_{m}$ is the average advancement offered by the $m^{th}$ node of the FCS, hereinafter referred to as node *m*. The advancements are organized in decreasing order, i.e., $a_{1}$ is the average advancement of the node of the FCS which brings the largest progress in the direction of the destination, $a_{2}$ is the average advancement of the node which brings the second largest advancement and so on. Thus, node *m* is the node with the $m^{th}$ largest advancement.

**Figure 4:**
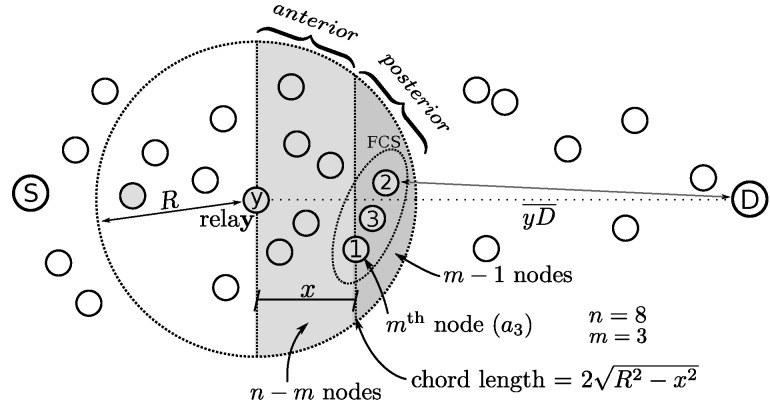
Example of calculation of the average advancement for forwarding candidate set (FCS) cardinality of three.

The calculation of $a_{m}$ is presented in Equations (10)–(13). Here we are considering that the distance from the current sender (relay *y*) to the destination dist(y,D) much larger than the radio range *R*, thus dist(y,D)≫R. This enables the approximation of the advancement by a chord in the circle centered by the sender with radios *R* and perpendicular to the line $\bar{yD}$, as depicted in Figure 4.

(10) $$a_{m}=\alpha \left(m,n\right)\int_{0}^{R}x\overset{m^{th}}{\overset{︷}{f(x)}}\overset{anterior}{\overset{︷}{{\left[1-\beta (x)\right]}^{n-m}}}\overset{posterior}{\overset{︷}{\beta {\left(x\right)}^{m-1}}}dx$$

(11) $$f\left(x\right)=\frac{2\sqrt{R^{2}-x^{2}}}{\left(\frac{\pi R^{2}}{2}\right)}$$

(12) $$\begin{array}{cc} \beta (x) & =\int_{x}^{R}f\left(z\right)dz=\frac{\int_{x}^{R}2\sqrt{R^{2}-z^{2}}dz}{\left(\frac{\pi R^{2}}{2}\right)}\\ \; & =\frac{2}{\pi }\left(arcos\left(\frac{x}{R}\right)-\frac{x}{R}\sqrt{1-{\left(\frac{x}{R}\right)}^{2}}\right) \end{array}$$

(13) α(n,m)=n︷mthpositionPossiblenodesfor×n−1m−1︷posteriorCombinationsfor.

Equation (10) integrates from zero to the radio range *R*. The integration variable *x* covers all possible advancements of the node with the $m^{th}$ largest advancement, which can be from zero to the radio range. An intermediary value of this variable is presented in the Figure 4. f(x) presents the probability density of finding the $m^{th}$ node over the chord in the position *x*. β(x) is presented in Equation (12). It calculates the probability of a node to be placed in the range area after the displacement given by *x*, i.e., being in the interval [x,R]. This area is identified as posterior in Equation (10). Since m−1 nodes should be in this area, β is powered by m−1 to calculate this probability.

The probability of a node to be located in the anterior area is 1−β(x), which is powered by n−m since the probability of encountering n−m nodes in this region is required. α(n,m), presented in Equation (13) returns the number of possible combinations derived from a scenario with the total of *n* nodes where the average advancement of the *m*-th node is in consideration. The *m*-th node can be any of the total of nodes, therefore the first term in the α equation is *n*. The possibility of different node selections for the posterior is given by n−1m−1, which is the second term. Since all not selected n−m nodes must be in the anterior, the last term in the multiplication is one.

In the example presented in Figure 4, $a_{1}$ is the advancement of node 2, $a_{2}$ is of node 3 and $a_{3}$ of node 1. The figure presents an example of calculation of $a_{3}$, i.e., m=3.

Equation (14) presents the estimation of *n* based on the node density η. Based on this value and the desired FCS cardinality, it is possible to appraise the average advancement reached by each hop in the network. Since the sender has the location of the destination, it is possible to assess the average number of hops necessary to reach it and to use this information to select the best initial delay to release the packet after the start of the preamble series.

(14) $$n=\eta \frac{\pi R^{2}}{2}$$

## 6. Experimental Results

The performance of our protocol was appraised and compared to state-of-the-art approaches using simulations.

### 6.1. Simulation Setup

In order to assess the efficiency of this innovative protocol, simulations were performed in the GrubiX Wireless Network Simulator, a redesign, and extension of ShoX [33]. For the simulations, it was adopted a radio model based on the IEEE 802.15.4, fixed transmission power, bidirectional links, and Free Space propagation model for isotropic point source in an ideal propagation medium. The radio range was configured to 40 m.

Sensor nodes were deployed randomly in the simulation arena and their locations form a Poisson process. The resulting connection graph is called a random geometric graph [34]. Nodes were static and exchanged position information at the beginning, therefore each node knows the neighbors’ positions. The proposed MAC protocol is combined with a geographic routing protocol, based on GPSR protocol [31].

The sleep/awake schedules of nodes were independent and random. In order to estimate the performance of our method, PAX-MAC performance was compared to the following protocols: X-MAC [3], X-MAC Anycast 2 [4], X-MAC Anycast 6 [4], GeRaF [29] and AGA-MAC [18]. X-MAC is a well known asynchronous MAC protocol whereas X-MAC Anycast 2 and 6 are improvements employing anycast with FCS cardinality of two and six proposed in [4]. GeRaF is a standard asynchronous anycast protocol. As already stated, AGA-MAC is an anycast asynchronous protocol which takes into account the packet size in order to reduce network latency.

In order to compare the different protocols, all parameters were set to the same values. The GeRaF protocol was adapted to avoid the use of continue packets which would decrease its performance. In this way, the behavior of GeRaF is the same as CMAC [28] without the use of threshold ($r_{0}$). Thus, from here on this protocol will be called GeRaF/CMAC.

Before a transmission takes place, the sending node performs a carrier sensing (1 ms). If no ongoing transmission is detected, the node starts to send a series of short-preambles, with a transmission time of 0.5 ms. Preambles are interleaved with a waiting time of 0.5 ms, when the radio is set to listening mode for eACK detection. When a neighbor node, member of the FCS, receives a preamble, it replies with an eACK message.

All protocols were evaluated in different scenarios. The parameters of each scenario are presented in Table 4.

It is important to remark that different packet sizes and network densities were employed for protocol assessment. Packet sizes were set according to the FCS cardinality of PAX-MAC. In this way, the packet transmission time is the same as the average time to find an awake node in the FCS. No ACK mechanism was used in our simulations to confirm data reception.

Each execution scenario comprises the transmission of one data packet from a source to a destination, whose distance, from the source comprehends 650 m in one scenario and 1300 m in another. Multi-hop transmission is employed to engender communication. In addition, the behavior of the protocols was also evaluated under the condition of concurrent traffic, i.e., multiple data flows.

In order to obtain statistically relevant results, each scenario was executed 120 times, with respect to a confidence level for confidence interval set to γ=1−α, α=0.05.

### 6.2. Results

The latency reduction achieved by the PAX-MAC is due to the separated data and preamble packets transmission. Depending on the situation, these two transmission events occur at the same time, but in different locations of the network. This section presents the evaluation of different aspects of the proposed protocol. Initially, the optimal initial delay is evaluated. In the sequence, network latency and energy consumption assessments are presented.

#### 6.2.1. Optimal Initial Delay

**Figure 5:**
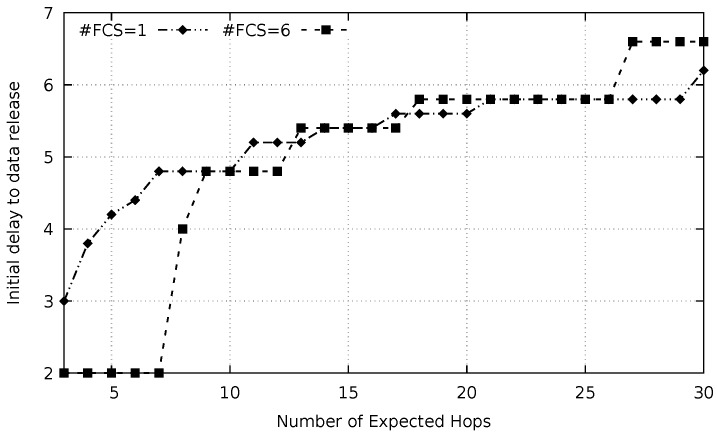
Optimal initial delay (in $t_{data}$) to release data as a function of the number of hops between source (S) and destination (D). Minimal initial delay limited in 2 data transmission time.

As explained in the previous section, there is an optimum value for the initial delay, which results in the lowest latency to send a message from a source node (S) to a destination node (D) considering there are *n* hops apart. Using the optimization function proposed in Section 5.4, this optimal value is calculated. Figure 5 presents the results of the optimization for two different conditions. In the first case, the chosen cardinality of the FCS was one. The second condition comprises a FCS cardinality of six nodes. The distance between the source and destination nodes varies from 3 to 30 hops. The initial delay is counted in data packet transmission duration ($t_{data}$).

It is important to remark that in all situations, the data packet duration ($t_{data}$) has a length equal to the time to transmit the average number of preambles per-hop (Equation (1)). For the optimization process, a resolution of $\frac{t_{data}}{5}$ was employed. Furthermore, a minimum value for the initial delay was set to $2\times t_{data}$.

It is possible to observe in Figure 5 that the optimal initial delay is higher for the case in which the #FCS = 1 compared to the case in which the #FCS = 6 when the distance between S and D is lower than 9 hops. For distances equal or greater than this (9 hops), the optimal initial delay values for both cases get closer to each other. Besides that, this value slowly increases with increments in the distance.

At first glance, one could suppose that the optimal initial delay should be $3\times t_{data}$ since preambles must go three hops ahead from the data packet. Results depicted in Figure 5 evince the misjudgment of this argument. This is a consequence of the fact that preamble propagation time is not constant but obeys a non-symmetrical probability density function.

Before start sending preambles, the sender node (S) calculates the optimum initial delay to send data. The calculation of this value depends on the distance of the sender to the destination node (D).

**Figure 6:**
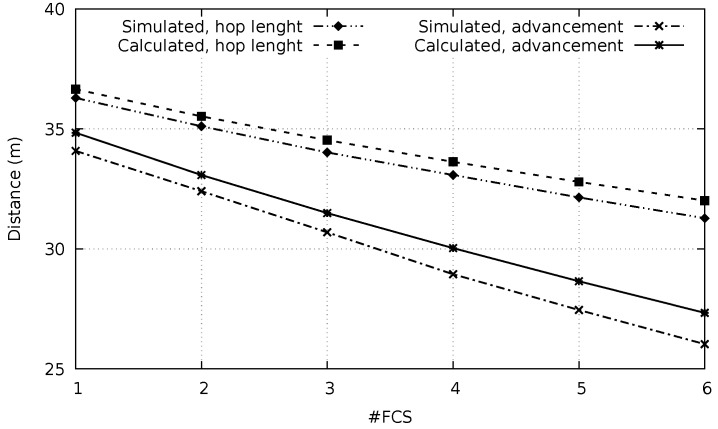
Average advancement and average hop length for distinct FCS cardinalities. $\eta =6\times 10^{-3}\frac{\mathrm{node}}{m^{2}}$.

The number of hops is calculated dividing the distance between *S* and *D* by the average advancement (adv), which is calculated using Equation (9). The average advancement reduces as the number of FCS members increases, as it can be observed in Figure 6. This figure presents both the theoretical and experimental results for the average advancement in a network with the density of $0.006\frac{\mathrm{nodes}}{m^{2}}$. The number of nodes in the FCS varies from 1 to 6. It is also possible to observe in the figure that the difference between the theoretical and the experimental results is lower than 6%.

The observed reduction of the average advancement as the number of the FCS members increases can be explained by the fact that the FCS members are chosen as those nodes which are able to provide the greatest progresses. As the number of FCS members increases, lower progresses have to be considered.

Figure 6 also presents theoretical and experimental results of the average hop length for one hop when different numbers of FCS members were considered. The theoretical results were calculated employing Equation (10). For that, the displacement *x* is substituted by the distance between the relay and the FCS member in this equation.

The average hop length is always greater than the average progress. Relays normally are not positioned close to the line defined by source (S) and destination (D), a fact that explains this difference. The average progress becomes closer to the hop average distance as the number of FCS members decreases. This is a consequence of the reduction of the search area of the FCS members.

The knowledge about the optimal initial delay showed in Figure 5 is used by the sender node to achieve the minimum latency. During the transmission, if a relay node identifies an imminent collision, it interrupts the preambles sending process until it is safe to restart this process, i.e., collision is avoided. Communication is restarted using the optimal delay as presented in this section.

**Figure 7:**
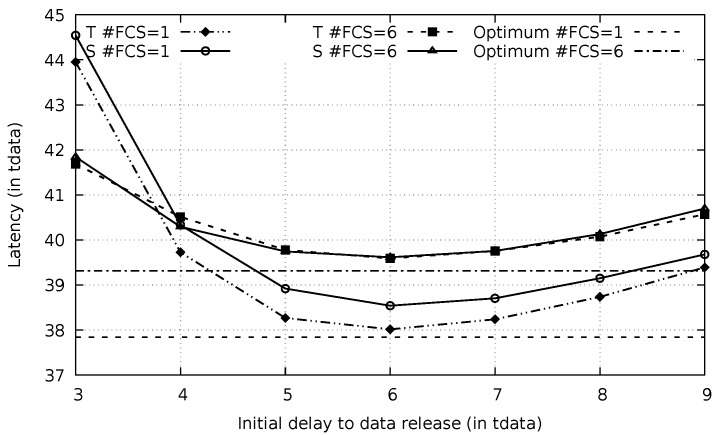
Latency as a function of initial delay to data release, for destination distant 30 hops from sender. Theoretical (T) and simulated (S) results for FCS cardinalities of 1 and 6. The two straight lines represent theoretical results for optimal (variable) initial delays.

Experiments were realized to verify that variable initial delay as presented in Figure 5 outperforms the use of a simple fixed initial delay. In each experiment, the data packet was released after a selected fixed initial delay and the communication latency was measured (for 200 repetitions). The results are presented in Figure 7 for #FCS = 1 and #FCS = 6. Initial delays ranging from $3\times t_{data}$ to $9\times t_{data}$ were probed. Destination (D) were 30 hops apart from source (S).

Besides experimental results, the figure also presents theoretical latencies calculated by the Equation (15), which is derived from Equation (8) for fixed initial delay δ values, in the same conditions of the simulated experiments. Notice that theoretical and simulated results are very close to each other (maximum deviation lower than 2%).

(15) $$e\left(n\right)=\delta +\sum_{i=1}^{n-1}ph\left(i,\delta \right)\cdot e\left(n-i\right).$$

Using the fixed δ, when the waiting time is set above the best value ($6\times t_{data}$), the total latency also increases for both scenarios. This can be explained by a smaller reduction of the imminent collision event which does not pay off the initial augmented waiting time. It is also possible to notice in Figure 7 that larger packets had a relatively smaller latency when compared to smaller packets (relative latency for #FCS = 1 is smaller than latency for #FCS = 6). The latency is measured in numbers of data packet duration ($t_{data}$) in this result. For example, for the optimal waiting time and $\eta =6\times 10^{-3}\frac{node}{m^{2}}$, a packet of 50% of $t_{cycle}$ has a latency corresponding to $37.8\times t_{data}=37.8\times 0.50\times 0.1$ s. On the other hand, a packet of 15% of $t_{cycle}$ has a latency of $39.4\times t_{data}=39.4\times 0.15\times 0.1$ s, when #FCS = 6. This can be explained by the average number of hops. Smaller packets require a larger number of nodes in the FCS, which reduces the average advancement and increases the number of hops to connect source to destination. With more hops, the normalized latency increases.

A better latency is obtained when variable initial delays, calculated by Equation (8), are employed. This is demonstrated in Figure 7 by the two horizontal lines, for #FCS = 1 and #FCS = 6. When an imminent collision between the data packet and the preamble is identified and the data sending restarts, it uses an initial delay corresponding to the number of remaining hops to reach the destination. For instance, in the case in which 6 FCS members are used for a 30 hops distance between *S* and *D*, the sender uses a delay of $6.6\times t_{data}$, as indicated in Figure 5. If an imminent collision is identified in the 18th hop, the corresponding relay node stops sending preambles and wait data arrival. In the following, this relay restarts the sending process towards destination but using an initial delay of $5.8\times t_{data}$. Using this varying initial delay allows a more significant latency reduction, as it is possible to be verified in the figure (horizontal lines).

#### 6.2.2. Latency Assessment

**Figure 8:**
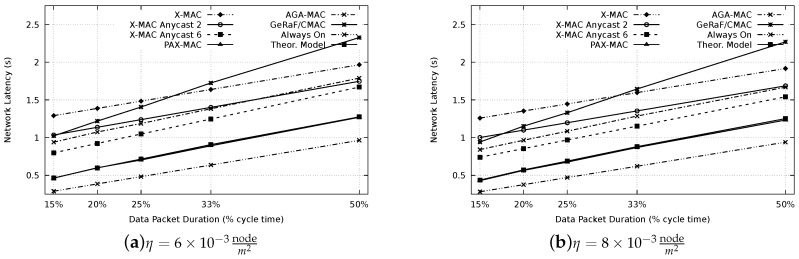
Latency for six different protocols compared to an always on network and the theoretical performance of the PAX-MAC. Source to destination distance set to 650 m. For all measurements, confidence interval shorter than $\left(\bar{x}+0.05,\bar{x}-0.05\right)$.

**Figure 9:**
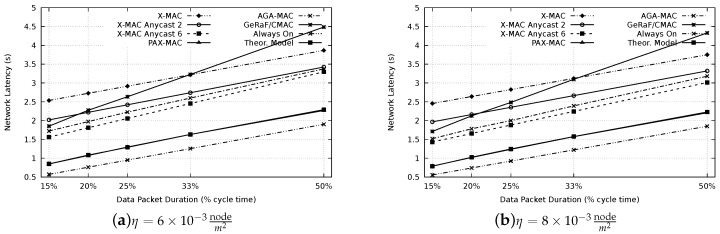
Latency for six different protocols compared to an Always On network and the theoretical performance of the PAX-MAC. Source to destination distance set to 1300 m. For all measurements, confidence interval shorter than $\left(\bar{x}+0.05,\bar{x}-0.05\right)$.

After setting up the optimal waiting period of the PAX-MAC, experiments to appraise the latency of our protocol and compare with the state-of-the-art were performed. Figure 8 presents the resulting latency for all protocols evaluated, for densities of $\eta =6\times 10^{-3}$ (a) and $\eta =8\times 10^{-3}\frac{\mathrm{node}}{m^{2}}$ (b). In this case, a distance of 650 m between source (S) and destination (D) was considered. Similar results for a distance of 1300 m are depicted in Figure 9. For comparison, we also included the latency for a network where nodes are maintained awaken all the time, therefore, preambles are not necessary to establish communication. This presents a performance boundary.

For the results, the following different packet sizes were considered: 15% of $t_{cycle}$ (#FCS = 6), 20% of $t_{cycle}$ (#FCS = 4), 25% of $t_{cycle}$ (#FCS = 3), 33% of $t_{cycle}$ (#FCS = 2) and 50% of $t_{cycle}$ (#FCS = 1). Each packet transmission time is selected according to the time expended by the average number of preambles for the corresponding FCS cardinality of PAX-MAC.

It is possible to notice that, for both densities, the results are similar, nevertheless the results are displaced to lower latency when density increases, as expected (less hops). As expected, larger packet sizes resulted in higher latencies, for each protocol.

The worst result for small packets was encountered in the X-MAC protocols. This can be explained by the fact that it does not use the anycast method, requiring, on average $\frac{N_{p}}{2}$ preambles per hop to forward packets. According to our previous analytical results [18], a small number of nodes in the FCS (one, in the case of X-MAC) does not present satisfactory result for small to medium packets, paying off just for very long messages due to the reduced number of hops. High FCS cardinalities reduce the latency when small packets are transmitted whereas FCSs with a small number of nodes present good performance for large packets (compared to the cycle time).

Thus, the performance of the GeRaF/CMAC is also influenced by this rule. Since the protocol uses all nodes in the range that bring improvement as FCS (large FCS cardinality for the densities used), it has a better performance for small messages. The result degrades when packet sizes are increased.

The performance of our previous AGA-MAC protocol is also presented in the results. The only state-of-the-art protocol which accomplished better results than the AGA-MAC was the X-MAC Anycast 6. This can be explained by the stability of the FCS cardinality in this method (always 6) whereas in the AGA-MAC, since positions of neighbors are not known, the FCS size varies. In some hops, a small number of the nodes may be part of the FCS, increasing considerably the number of preambles needed to advance to the next hop. Moreover, the average advance is slightly smaller when the FCS size is not fixed.

Even possessing a fixed FCS size (two), the X-MAC Anycast 2 protocol presented a lower performance when compared with AGA-MAC (and the X-MAC Anycast 6). This result shows that, for the appraised parameters, an FCS cardinality of six is more appropriate for the class of X-MAC Anycast protocols.

The results also showed that our novel PAX-MAC protocol performs well. For all packet sizes and network densities, the transmission latency was considerably smaller than for all other exercised protocols. This is true for both distances scenarios: 650 m and 1300 m. Our protocol outperforms the best state-of-the-art (X-MAC Anycast 6) in at least 20% for all packet sizes and considered scenarios. The performance of the proposed protocol can be better recognized when contrasted with a network without duty-cycle (i.e., the nodes are aways on). The distance, in latency, of our protocol to this theoretical limit is less than its distance to any other examined stat-of-the-art protocol.

#### 6.2.3. Latency with Concurrent Flows

**Figure 10:**
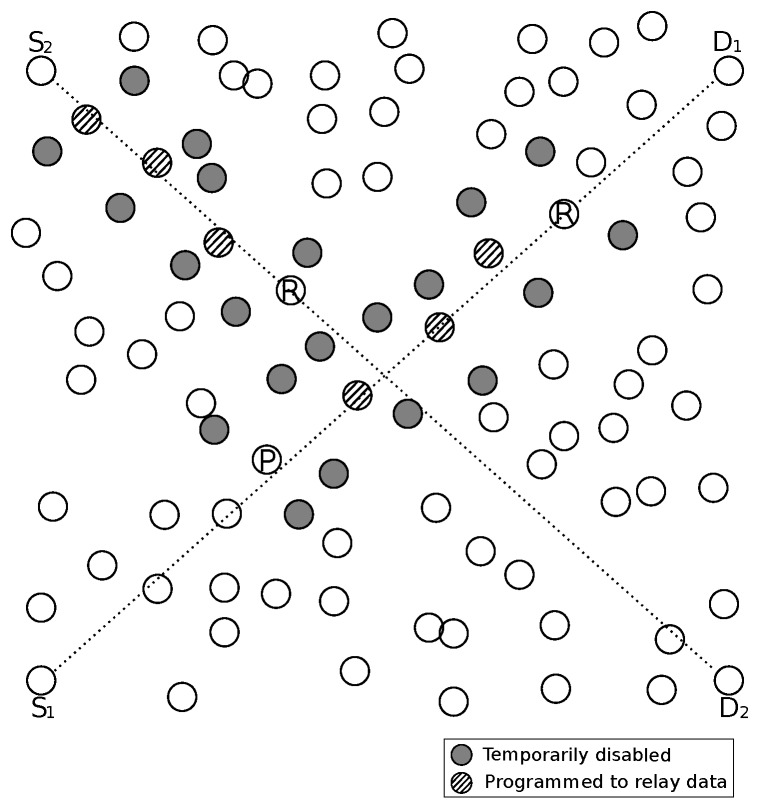
Simulation scenario with two concurrent data flows.

In order to appraise the behavior of our protocol in scenarios with high communication traffic, experiments with concurrent communication flows were accomplished. The layout of Figure 10 was employed in order to provoke concurrent communications with contention for the nodes in the crossing region. The nodes $S_{1}$ and $S_{2}$ are the sources of two different concurrent communications and nodes $D_{1}$ and $D_{2}$ the respective destinations. Eventually, a packet from one flow may collide with a packet from the concurrent one. It is a collision inter different flows, here denominated inter-collision. This is different from the imminent collision which occurs between preambles and data packet from a single flow.

The figure also illustrates the mechanism used by the PAX-MAC to avoid the inter-collision between packets of competing ongoing transmissions. In the example of transmission depicted by the figure, nodes labeled by *R* are prospecting the path from the source to the destination by issuing preambles—RTS packets. Nodes denoted by *P* are carrying at the moment the data packet. Hatched nodes have been selected to form the path towards the destination and are programmed to receive and relay the data packet.

As already stated, the inter-collision avoidance mechanism for concurrent communication flows comprises temporarily disabled nodes which prevent the RTS packets from different flows to interfere in data packet transmission of the current one. The temporarily disabled nodes are programmed to avoid the execution of the duty-cycle until the data packet has successfully being transmitted by nearby relay nodes. The disabled nodes act as a barrier, they do not respond to the RTS packet from other communication flows, preventing possible inter-collisions. They are represented in the figure as gray nodes. These nodes enter the disable condition when hearing RTS packets not addressed to them.

In the example depicted by Figure 10, the node *R* in the path $S_{2}\to D_{2}$, will not receive an answer (CTS packet) from its FCS members since they are temporarily disabled. This condition is kept until the data packet *P* has transversed the nearby nodes.

The setup of the simulation is a subset of that depicted in Table 5, since only 650 m distance from source to destination was considered, as soon as the node density of $8\times 10^{-3}$. Two data packet duration were tested, $t_{data}=50\%t_{cycle}$ and $t_{data}=15\%t_{cycle}$, corresponding to FCS cardinalities 1 and 6 in PAX-MAC protocol. Initial waiting time was set to k=6. Each simulation comprises two independent message flows as depicted in Figure 10, each one initiated by a source node ($S_{i}$) deployed in a corner of a square area and flowing towards the corresponding destination ($D_{i}$) in the opposite corner. All the remaining nodes are randomly deployed in the area. The starting time of each flow is randomly chosen by the sender node within a range from zero to $t=5.t_{cycle}$.

We evaluated the protocols in two different modes, denominated as persistent mode and non persistent mode. In non persistent mode, the sender node transmits RTS packets in order to achieve an awaken neighbor. This process is repeated for a period equal to the cycle time. If no answer (eACK) is sent back, sender node stops sending RTS packet and data packet is discarded. If persistent mode is employed, sender node restarts to send RTS packets when no answer is heard after a cycle time. This retrying process is repeated for a predefined number of times. In our simulations, if no answer is heard after three attempts, the data packet is discarded. The protocols GeRaF/CMAC, X-MAC, X-MAC Anycast 2 and X-MAC Anycast 6 were adapted to perform the retrying process, since this functionality is not present in original ones. This process is done in the same mode AGA-MAC does, after sending all the preambles in a cycle time without receiving an answer, the sender node restarts to send a new series of preambles. The behavior of PAX-MAC is slightly different from the others, since the imminent collision condition is achieved before the last retry process finishes. Then, the sender node will stop sending preambles and wait for data packet arrival, after what it takes the role of the source node. Another difference is that inter-collisions with data packets are avoided in PAX-MAC when the nodes nearby the relays of a flow are disabled.

**Figure 11:**
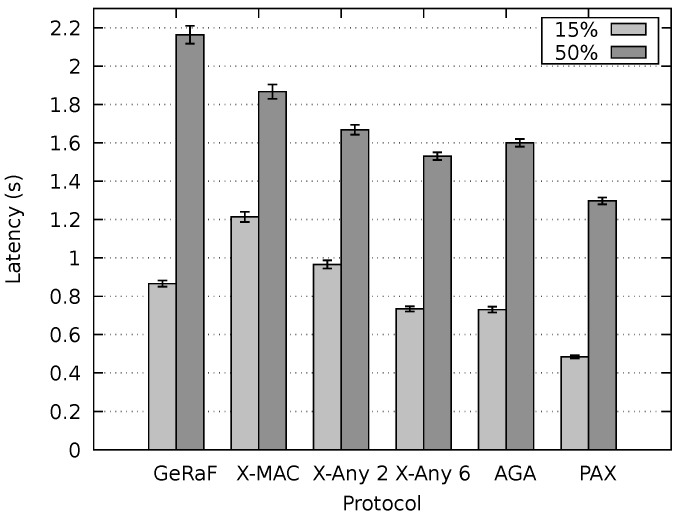
End-to-end latency for concurrent flows transmitted in non-persistent mode, for data packet durations $t_{data}=50\%t_{cycle}$ and $t_{data}=15\%t_{cycle}$, $\bar{SD}=650$ m.

After setting up the environment to inspect the behavior of our protocol under concurrent flows and optimal delays, experiments to appraise the latency of our protocol and compare it with the state-of-the-art were executed. Figure 11 shows the resulting latency for non-persistent mode. The message duration was 15% and 50% of $t_{cycle}$ and the sender and receiver were placed 650 m apart. As expected, the average latency for the small messages (15% of $t_{cycle}$) was considerably lower than for large messages for all protocols. Our PAX-MAC protocol performs better than all others in this experiment, presenting the lowest latency for both messages sizes. For small messages, the gain was similar to when no other traffic was present in the network (single flow scenario).

For large packets (50% of $t_{cycle}$), the protocols X-MAC Anycast 2, X-MAC Anycast 6 and AGA MAC present a similar results, performing considerably worse than our PAX-MAC protocol. The X-MAC and GeRaF/CMAC protocols exhibit the largest latency in this experiment. These results are similar to the ones obtained in the single flow scenario.

**Figure 12:**
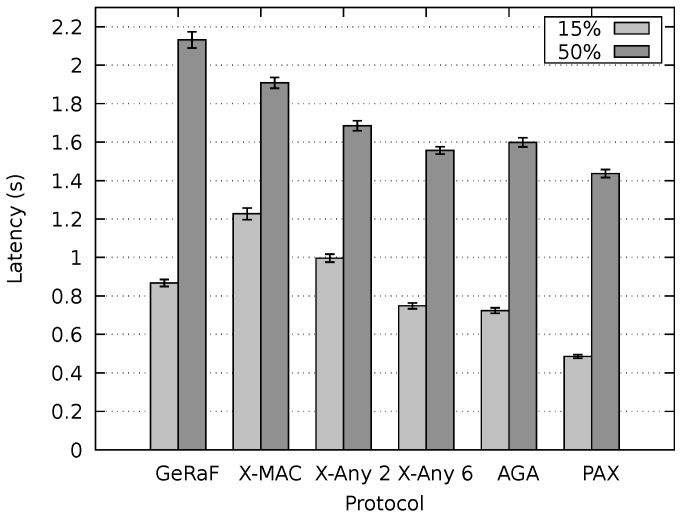
End-to-end latency for concurrent flows transmitted in persistent mode, for data packet durations $t_{data}=50\%t_{cycle}$ and $t_{data}=15\%t_{cycle}$, $\bar{SD}=650$ m.

Figure 12 presents the results for the persistent mode. It can be noticed that, for the majority of cases, the outcomes are very similar to the previous ones. The advantage of employing the retry mechanism can be observed in the packet delivery success rate for large (50% of $t_{cycle}$) packets.

**Figure 13:**
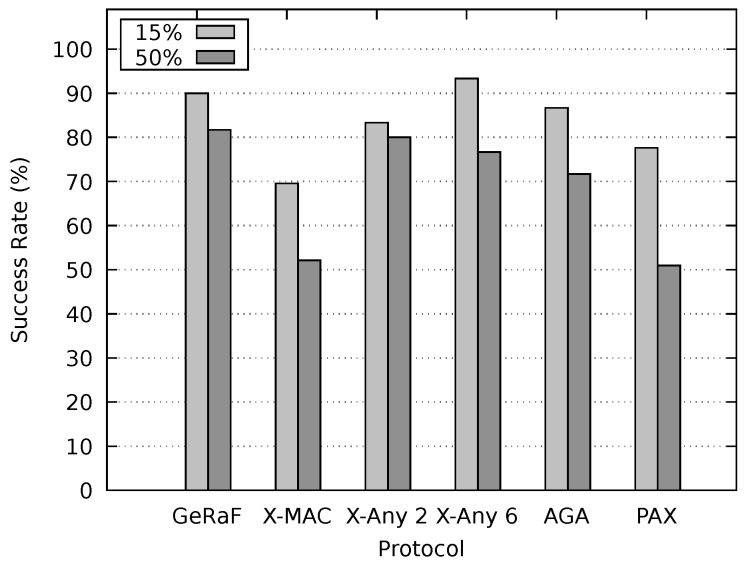
Packet delivery success rate for concurrent flows transmitted in non-persistent mode, for data packet durations $t_{data}=50\%t_{cycle}$ and $t_{data}=15\%t_{cycle}$, $\bar{SD}=650$ m.

**Figure 14:**
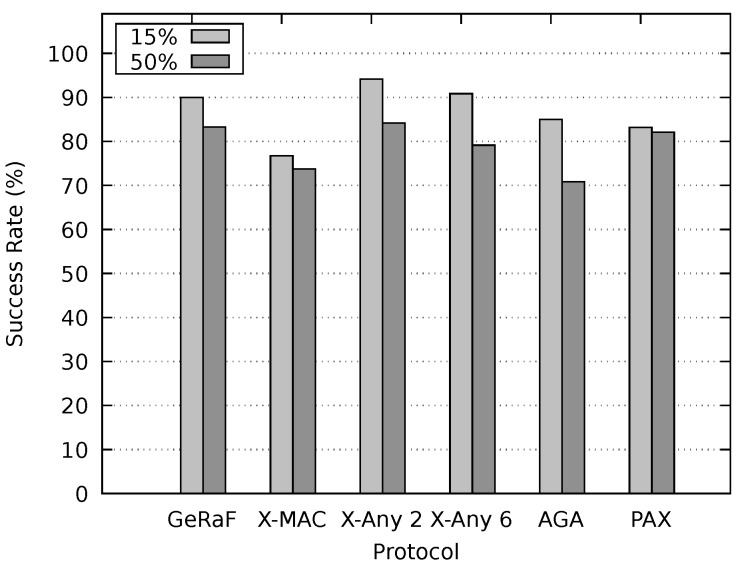
Packet delivery success rate for concurrent flows transmitted in persistent mode, for data packet durations $t_{data}=50\%t_{cycle}$ and $t_{data}=15\%t_{cycle}$, $\bar{SD}=650$ m.

Figure 13 and Figure 14 present the packet delivery success rate for all assessed protocols considering the non-persistent and persistent modes respectively. When the persistent mode was employed, the packet delivery success rate was higher for the X-MAC and X-MAC Anycast 2 for all message sizes and PAX-MAC with large packets. This fact is associated with the small cardinality of the FCS for these configurations. Moreover, for all configurations, for a given protocol, the success rate was higher for packet duration of $t_{data}=15\%t_{cycle}$ than for the large packets. This can be explained by the fact that larger packets are using network resources for a longer time, increasing the probability of inter-collisions.

For small packets, our protocol presents a slightly smaller success rate than other protocols at the same time as providing a substantial latency reduction. For packet duration of $t_{data}=50\%t_{cycle}$, our protocol was able to deliver about 50% of data packets in the non-persistent mode. This can be explained by the large time interval of blocked resources in the network, caused by the separated transmission of preambles and data and also by the mechanism of temporarily disabling nodes to avoid inter-collision and packet loss. Without retry, a flow that is blocked by the passage of another with its temporarily disabled set of nodes can not retry to find a awaken node in the FCS when the set of preambles come to an end. Thus, the data packet does not arrive at the destination, decreasing the success rate. Nevertheless, this performance is highly increased when the persistent mode is employed, yielding the highest success rate among all other protocols appraised.

Explanation for this positive performance lies in the imminent collision mechanism. When a flow is blocked by temporarily disabled nodes, the preambles are sent until the maximum count is achieved. Then, the retry mechanism is employed: the sequence of preambles is restarted. Eventually, the imminent collision event happens: transmission of preambles is canceled, node waits until the data packet arrives and then starts preambles again. This procedure consumes a considerable amount of time. After that period of time, when preambles are restarted, data packet of the other flow has already been transmitted, releasing the shared region, thus allowing next relay to be found. The protocol continues its operation normally and eventually, data packet is delivered to destination.

#### 6.2.4. Multiple Simultaneous Flows

Next experiments aimed to observe latency and delivery rate in scenarios with several simultaneous transmissions. Nodes are randomly deployed in a squared area with 1000 m long side, which locations form a Poisson process. During a defined sending interval Δ, *f* pair of nodes were randomly selected. Each source node $S_{i}$ sends a message to destination node $D_{i}$, where i=1...f. Each sending process starts at a random time point inside the interval Δ. For this appraisal, the configuration presented in Table 5 was selected. Other parameters were set as presented in Table 4. The average Source to Destination distance can be calculated as 0.52×1000=520 m, as a Square Line Picking.

**Figure 15:**
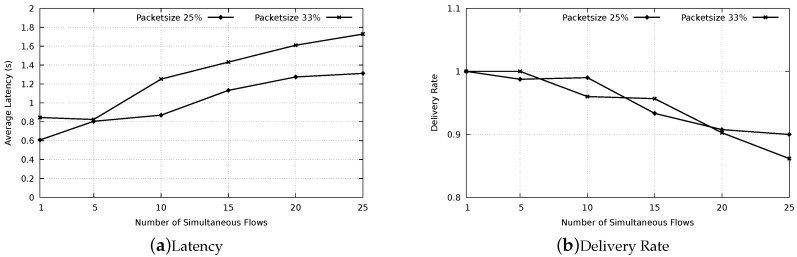
End-to-end latency (**a**) and delivery rate (**b**) with 1,5,10,15,20,25 different flows dispatched within a Δ time interval.

Experiments were run twenty times for each configuration and the average values were computed. Figure 15a presents end-to-end latency as function of the number of flows for both packet sizes ($0.25t_{cycle}$ and $0.33t_{cycle}$). In both cases, end-to-end delay has a sensible rise with the number of flows. This was expected due to the increased number of collisions and transmissions restarts. It is important to remark that an ACK packet was introduced to detect data loss. Every time a message is transmitted and the ACK is not received, a random length back-off ranging from $1t_{cycle}$ to $2t_{cycle}$ was carried out. Three attempts to transmit the data message were allowed. Dropped packets were not taken into account for latency calculation.

Figure 15b presents delivery rate as function of the number of flows for both packet sizes of $0.25t_{cycle}$ and $0.33t_{cycle}$. The increased number of simultaneous messages yields in a lower delivery rate. It is important to remark that a limit of three attempts to transmit the data message was set. After this, the message is dropped. If more retries were allowed, an increased performance would be observed in the delivery rate, however, the latency would suffer even more.

Our protocol was designed for light traffic WSNs. Notwithstanding, in situation with network stress, as assessed in these experiments, it achieved a high deliver rate with a penalty in latency. This penalty is compensated by its high speed compared to other protocols, as presented in Figure 8.

#### 6.2.5. Energy Consumption Assessment

Comparisons related to energy consumption of our protocol and the other five protocols were performed, in the scenario with a single flow. The results count only the energy that was spent to send a data packet from the source to a destination 650 m away and to a destination 1300 m away. They do not consider the energy consumption due to the ordinary network operation, i.e., the stand by, in which the sensor nodes periodically wake up to perform the carrier sense. The energy consumption due to stand by depends on the total number of nodes in the network. It is considered the same for all tested protocols, except for the Always On.

For all protocols, the energy spent in each Source to Destination communication is calculated by:

(16) $$E=P\sum_{h=1}^{N_{h}}\left[t_{cs}+p_{h}\left(t_{eACK}+t_{pre}\right)+t_{eACK}+2t_{data}\right]$$

where *E* represents the energy spent in mJ, $t_{cs},\;t_{eACK},\;t_{pre}$ and $t_{data}$ are given in Table 4, $p_{h}$ is the number of preambles transmitted by the relay node in hop *h*, and $N_{h}$ is the total number of hops. *P* is the power a node dissipates in awake state, in mW. It was considered that radio consumption is the same in idle listening, transmission and reception. This power is multiplied by the sum of the total time of transceiver activity, modeled by the rest of the equation. This is the sum of the transceiver operating time in both sender and receiver modes for each hop (from node 1 to $N_{h}$, where 1 is source node and $N_{h}$ is destination). For hop *h*, sender node performs a carrier sense ($t_{cs}$) and sends $p_{h}$ preambles. The total time of consecutive preambles is calculated by the sum of the preamble sending time with the waiting time for eACK ($t_{pre}+t_{eACK}$). After the preamble $p_{h}$, the next relay sends an eACK packet ($t_{eACK}$). Data packet is then transmitted by the sender and received by the next relay. In energy computation, both events are considered ($2t_{data}$).

**Figure 16:**
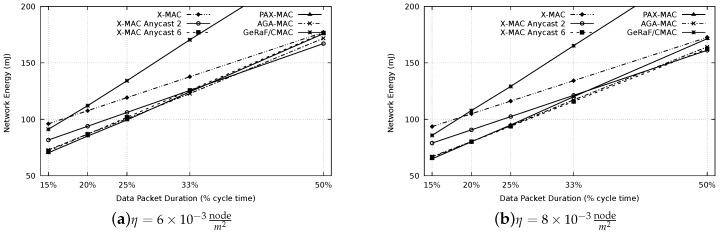
Energy consumption for the 6 different protocols, with the destination 650 m distant from the source. For all measurements, confidence interval shorter than $\left(\bar{x}+5,\bar{x}-5\right)$.

Figure 16 depict the average energy consumption of all executed protocols for both network densities. Results for the always-on are not shown in these graphs because its energy consumption is much higher than the other protocols since it is not a duty-cycled protocol. For other tested protocols it was used a duty-cycle of 1%. Thus, all of them present energy consumption 100 times smaller than the always-on.

It is possible to observe in Figure 16 that GeRaF/CMAC presented an energy consumption higher than the others for almost all data packet sizes, for both network densities. GeRaF/CMAC presented lower consumption only in relation to the standard X-MAC for small packets (15% of the cycle time). Including in the FCS all the nodes that may promote advancement towards destination has as consequence the reduction in average packet advancement. For the other tested protocols, the average advancement is always superior to the one presented by GeRaF/CMAC. This behavior is explained by the fact that only the neighbors giving the most significant advancements take part in FCS. As a consequence, more hops are needed to reach destination when using GeRaF/CMAC. When large data packets are transmitted, the additional energy consumption in each hop becomes significant.

Besides GeRaF/CMAC, it is possible to observe that the energy consumption of all other protocols converge as the size of the packet increases. This behavior is justified by the fact that a significant part of the energy consumption is due to the transmission of long data packets. For data packets bigger than 50% of cycle time, the energy spent in transmissions and receptions of these packets is always greater than the energy spent in control messages exchange (preambles and eACK).

The results achieved by PAX-MAC for energy consumption were lower for all tested scenarios ($\eta =6\times 10^{-3}$ and $\eta =8\times 10^{-3}\frac{\mathrm{node}}{m^{2}}$) for the case of small data packets (15% of the cycle time). X-MAC Anycast 6 and AGA-MAC presented the best energy behavior, in average. Only for the data transmission duration of 50% of cycle time ($t_{data}=0.5t_{cycle}$), X-MAC Anycast 2 surpassed all the others in both scenarios. The energy consumption of PAX-MAC was close to the other protocols. In the worst case, it was only 6% above AGA-MAC for the tests performed with low and high-density scenarios. It is possible to observe a similarity between Figure 16a,b, in which the energy consumption for the low-density scenario was always higher than the consumption in the high-density scenario. However, the difference in energy consumption was always lower than 9%. The differences are due to the smaller number of hops in the high-density scenario.

## 7. Conclusions

Anycast protocols were proposed for reducing the sleep-delay problem in asynchronous wireless sensor networks. They exploit the path redundancy in densely deployed networks by selecting a group of nodes as good candidates to forward a message. This group of nodes is denominated FCS (Forwarding Candidate Set). The anycast protocol PAX-MAC proposed in this work splits the communication process in two phases. In the first one, the source node transmits a series of short preambles ahead in order to rendezvous with a next-hop node. This process is repeated for a number of hops before starting the second phase. In the latter stage, the source node releases data packet. With this process, simultaneous transmissions of preambles and data are explored, achieving further latency reduction. All the nodes belonging to the route between source and destination receive a data transmission schedule from the preamble packet. Then, these nodes stagger their wake-up time to receive and forward data packet with the shortest delay.

In PAX-MAC, the cardinality of FCS is selected to promote the same propagation speed of a preamble series and a data packet. In average, the distance between the node transmitting preambles and the one sending data is kept constant. In the case of lateness of preamble propagation, PAX-MAC presents a procedure to handle imminent collisions.

Our protocol was compared to X-MAC, AGA-MAC and an enhanced version of GeRaF based on CMAC. Two versions of the AnyMac protocol were also investigated, denominated here as X-MAC Anycast 2 and X-MAC Anycast 6. Simulations demonstrated that the proposed protocol outperforms all of them in latency for different network densities. In this evaluation, differently from previous ones, data packet sizes were taken into account. In comparison to the protocols assessed, for different inspected scenarios, our method presented results closer to a network that maintains nodes always on.

The PAX-MAC energy expenditure was similar to the best one for all scenarios. In worst case, it spent 6% more energy than the best one. This is a small price to pay inasmuch as a gain of 20% in latency was obtained.

## Figures and Tables

**Table 1:** List of all MAC states present in the state machine.

State	Description
Sl—Sleeping	Node is inactive
PC—Probing channel	Channel is probed for a preamble packet
CS—Carrier sensing	Channel is probed for activity
SP—Sending preamble	Sender node is transmitting a preamble
WR—Waiting reply packet	Sender node is waiting for a reply packet
Wt—Waiting τ	Node is waiting a determined period before sending data packet
SR—Sleeping for receive data	Node is sleeping until the time to receive data
SM—Sending Message	Node is sending data packet
RM—Receiving Message	Data packet is received
VF—Verifying FCS	Verifying whether node is a FCS member

**Table 2:** List of all transitions in the state machine.

Transition	Description
1	Sleep timeout
2	Probing channel timeout. No activity detected
3	Preamble received
4	Node is not member of FCS
5	Node is member of FCS
6	Channel busy
7	Channel free
8	Preamble sent. Final destination
9	Preamble sent. Relay node
10	Reply to preamble not detected
11	Reply to preamble detected
12	Time for data reception achieved
13	Message received by Destination node *D*
14	Message received by a relay node
15	Imminent collision timeout
16	Time for data reception achieved
17	Message received
18	Message pending for transmission
19	Channel free
20	Preamble sent
21	Reply to preamble not detected
22	Reply to preamble detected
23	τ timeout
24	Message sent

**Table 3:** List of all variables in the mathematical model.

Variable	Description
τ	Initial delay, the waiting time between starting preambles and sending DATA
$N_{p}$	Maximum number of preambles in a cycle time
*i*	Number of Preambles spent until an awake node is found in current hop
*k*	Number of Preambles spent since the first one until an awake node is found in hop *n*
*q*	Probability function of using *i* preambles to encounter an awake FCS member
*p*	Probability of advancing *n* hops with *k* preambles when FCS cardinality is *v*
*r*	Average number of preambles necessary to encounter an awake node in the FCS
δ	Initial delay to release data packet given in number of *r*
*v*	FCS cardinality
*n*	Target number of hops to send a message without collision
$ps_{n}$	Probability of successful transmission of a packet through *n* hops without collisions
tl	Average total latency, measured in data packet length, to relay a packet *n* hops
*e*	Additional latency brought by the reservation of the channel and possible collisions
ph	Probability of colliding (imminent collision) on hop *i*
adv	Average advancement in each hop
*a*	Average advancement offered by a FCS member
α	Number of possible combinations
β	the probability of a node to be placed in the range area after the displacement given by *x*
*R*	Radio range
η	Average density of nodes in the WSN area
*x*	Advancement towards destination

**Table 4:** Parameters used for simulation experiments.

Symbol	Parameter	Values
*R*	Radio range (m)	40
*P*	Transceiver power (mW)	60
$\bar{SD}$	Source−destination distance (m)	650, 1300
η	Node density ($\frac{\mathrm{node}}{m^{2}}$)	$6\times 10^{-3}$, $8\times 10^{-3}$
$N_{p}$	Maximum number of preambles	98
$t_{cycle}$	Cycle time (s)	0.1
*v*	FCS cardinality	1, 2, 3, 4, 6
$t_{data}$	Data transmission duration ($\%t_{cycle}$)	50, 33, 25, 20, 15
$t_{pre}$	Preamble transmission duration (s)	$0.512\times 10^{-3}$
$t_{eACK}$	eACK transmission duration (s)	$0.512\times 10^{-3}$
$t_{cs}$	Carrier sense duration (s)	$1.024\times 10^{-3}$
τ	Initial waiting time (s)	$k\cdot r\left(v\right)\cdot \left(t_{pre}+t_{eACK}\right)$; k=3,4,5,6,7,8,9
*h*	Threshold (for AGA-MAC)	0.5

**Table 5:** Parameters used for simulation with multiple flows.

Symbol	Parameter	Values
*f*	Number of simultaneous flows	1,5,10,15,20
$\bar{SD}$	Source−destination distance (m)	Variable, in average 0.52×1000 m, Square Line Picking
η	Node density ($\frac{\mathrm{node}}{m^{2}}$)	$6\times 10^{-3}$
$t_{data}$	Data packet duration ($\%t_{cycle}$)	33, 25
τ	Initial Waiting Time (s)	$k\cdot r\left(v\right)\cdot \left(t_{pre}+t_{eACK}\right)$; k=6
Δ	Interval within senders start communication	10 s
